# Integrating Economic Theory, Domain Knowledge, and Social Knowledge into Hybrid Sentiment Models for Predicting Crude Oil Markets

**DOI:** 10.1007/s12559-023-10129-4

**Published:** 2023-03-20

**Authors:** Himmet Kaplan, Albert Weichselbraun, Adrian M. P. Braşoveanu

**Affiliations:** 1 University of Applied Sciences of the Grisons, Chur, Switzerland; 2 webLyzard technology, Vienna, Austria; 3 Modul Technology GmbH, Vienna, Austria; 4 Modul University Vienna, Vienna, Austria

**Keywords:** Affective models, Language models, Embeddings, FinBERT, Affective classification, Financial sentiment analysis

## Abstract

For several decades, sentiment analysis has been considered a key indicator for assessing market mood and predicting future price changes. Accurately predicting commodity markets requires an understanding of fundamental market dynamics such as the interplay between supply and demand, which are not considered in standard affective models. This paper introduces two domain-specific affective models, CrudeBERT and CrudeBERT+, that adapt sentiment analysis to the crude oil market by incorporating economic theory with common knowledge of the mentioned entities and social knowledge extracted from Google Trends. To evaluate the predictive capabilities of these models, comprehensive experiments were conducted using dynamic time warping to identify the model that best approximates WTI crude oil futures price movements. The evaluation included news headlines and crude oil prices between January 2012 and April 2021. The results show that CrudeBERT+ outperformed RavenPack, BERT, FinBERT, and early CrudeBERT models during the 9-year evaluation period and within most of the individual years that were analyzed. The success of the introduced domain-specific affective models demonstrates the potential of integrating economic theory with sentiment analysis and external knowledge sources to improve the predictive power of financial sentiment analysis models. The experiments also confirm that CrudeBERT+ has the potential to provide valuable insights for decision-making in the crude oil market.

## Introduction

The efficient market hypothesis (EMH) assumes that market prices incorporate all relevant information available to the public [1]. New events or newly available information, therefore, triggers price changes, although factors such as market cycles, contagions from other equities (e.g., movements in stocks that affect other markets, as witnessed during the infamous March 2020 crash), and changes in people’s sentiment towards specific equities can still affect market movements. Doyne et al. [2], for instance, observed that liquidity fluctuations caused by changes in supply and demand or other less predictable events considerably impact market prices.

Sentiment has become a key metric for assessing and predicting market performance. It is treated as an indicator that is used alone or together with other metrics for describing the current market mood, determining the potential and the most likely direction of price changes, and estimating future market prices. For instance, in December 2019, the ongoing trade dispute between the USA and China led to a negative shift in investor sentiment towards companies with significant exposure to the Chinese market, resulting in a decrease in stock prices for these companies.

Most approaches use standard affective models for computing market sentiment, which seems problematic since trading usually requires specialized knowledge. Stock markets, for instance, involve securities that generally represent equity in real companies, and derivatives such as futures contracts which make assumptions on future equity prices. Both instruments require knowledge of company fundamentals (e.g., EBITDA ratios, key people, financial reports), the markets in which the company operates, and technical analysis.

Macroeconomic and microeconomic news typically influences stock prices. For example, in November 2019, the signing of the United States-Mexico-Canada Agreement (USMCA) led to a positive change in consumer sentiment, resulting in an increase in stock prices for companies in the agriculture and manufacturing sectors. Standard affective models which have been slightly adapted to operate in the stock market seem to capture people’s feelings on market developments quite well, and are capable of distinguishing positive assessments (e.g., “AAPL went up.”) from negative (e.g., “Oil settled at a negative price.”), neutral (e.g., “The market moves in waves.”), or ambivalent (e.g., “Horrible crash, but quick recovery.”) ones.

For commodities (e.g., oil, iron ore, coffee), in contrast, factors such as geopolitics (e.g., which countries produce the respective commodities), storage, and the interplay of supply and demand play a major role. Standard sentiment indicators might even be misleading for commodities since a positive event can lead to either higher (e.g., caused by higher demand) or lower (e.g., due to more supply) prices.

For instance, a headline covering the disruption of a production site due to an accident (e.g., “Major explosion and fire at an oil refinery, casualties feared.”) would typically indicate a decrease in the overall oil supply and consequently cause oil prices to rise. Yet, current sentiment classifiers fail to perceive news of an accident, as a positive sentiment. These models are trained on large datasets which are likely to associate accidents with a negative sentiment. Standard sentiment classifiers, therefore, frequently fail to understand the event’s impact on the oil price, since they have not been designed to distinguish a text’s sentiment from its impact on a commodity’s price.

Consequently, accurately capturing the market’s current state and predicting market movements requires models that incorporate knowledge of fundamental market theories.

The research presented in this paper builds upon CrudeBERT, a domain-specific affective model that has been fine-tuned by applying Adam Smith’s theory of supply and demand to crude oil markets. Although CrudeBERT is well-suited for predicting the direction of price movements, it is currently incapable of quantifying them.

Extensive experiments identified the following feature sets that help in addressing this shortcoming:

- **Common knowledge** on the named entities mentioned in news headlines aids in assessing the importance of the covered events;
- **Numerical clues** extracted from the numbers mentioned in headlines yield information on an event’s strength; and
- **Social knowledge** from Google Trends quantifies an event’s coverage and provides another proxy metric for its potential impact.

Blending these features into CrudeBERT yielded CrudeBERT+, a hybrid approach that uses language models, economic theory, and external knowledge to predict price movements in the crude oil market.

The main contributions of this paper are

1. The introduction of CrudeBERT+, a domain-specific affective model for financial sentiment analysis (FSA) that uses market theory for estimating crude oil prices, and draws upon external knowledge from sources as varied as Wikipedia, World Population Review,^1^ and Google Trends to improve the model’s training and prediction steps;
2. Assembling a publicly available benchmarking dataset of news coverage and its impact on WTI crude oil futures;
3. An extensive study of the model’s predictive power which includes a thorough evaluation and an ablation study.

The rest of this article is structured as follows. The “Related Work” section provides an overview of related work and the current state of the art. Afterward, the “Problem Statement” section motivates the need for domain-specific affective models, to accurately predict crude oil markets. The “Method” section introduces the proposed method, which is followed by a comprehensive evaluation (“Evaluation” section) and discussion of the results (“Discussion” section). The paper concludes with an outlook and conclusions presented in the “Outlook and Conclusion” section.

## Related Work

Articles on stock price prediction use techniques as varied as univariate or multivariate forecasting [3], sentiment analysis [4], random walks [5], fractals and chaos theory [6], fundamental and technical analysis [7], and even news and social media analysis [8]. Besides what is published, there is also a wide array of proprietary systems that support retail traders or institutions, and which may never be described in the literature. Stock market prediction is one of the most competitive research areas, and obtaining a comprehensive snapshot of its current state of the art is almost impossible. Consequently, this section focuses mostly on research that includes sentiment and other natural language processing (NLP) features in the context of FSA.

A survey from Gu et al. [9] suggests that trading and price-based methods together with deep learning yield good results, surpassing traditional features such as text and sentiment. Hu et al. [10] review neural networks used for Forex and stock market prediction and discover that sentiment is rarely used in the papers submitted to top journals. Although this may have been the case for articles considered in that particular survey, the following discussion of the state of the art demonstrates that sentiment has been used extensively in recent literature.

Mahata et al. [11] suspect that due to the disruptions caused by the early 2020 coronavirus-induced market crash, some models described in the literature may no longer perform well in today’s markets. Of particular concern is the lack of representative data for the pandemic period, as data source variability can be a serious source of confusion for machine learning (ML) algorithms [12]. For instance, in March 2020, the spread of COVID-19 led to a negative shift in consumer sentiment, resulting in decreasing stock prices in a volatile market environment particularly in the tourism sector and futures of energy commodities. Nine months later, the approval of multiple COVID-19 vaccines led to a positive shift in consumer sentiment, resulting in an increase in stock prices for companies, especially in the healthcare sector.

The importance awarded to news and sentiment has also increased considerably during the pandemic, as showcased in a recent study that analyzed social media to understand the changes in the public’s behavior [13].

### Affective Models

While some models presented in this section have not been used for market prediction, it is necessary to briefly review them to fully understand the discussion on sentiment indicators’ affective models. This section firmly focuses on Transformer architectures that were used for such tasks. Many affective classification models have been introduced in the past three decades: Ekman [14], Plutchik’s Wheel of Emotions [15], the Circumplex Model of Affect [16], the Hourglass of Emotions [17], and its revised version [18]. These models generally distinguish between basic and derived emotions. Plutchik’s popular model [15] was the original gold standard for decades due to its combination of a workable structure (derived from the eight basic emotions: joy, trust, fear, surprise, sadness, disgust, anger, and anticipation) with different degrees of expression along the covered affective categories. The *Hourglass of Emotions* [17] identifies four categories (pleasantness, attention, sensitivity, and aptitude) and their activation scales (e.g., *pleasantness* can have various activation levels between *joy* and *sadness* like *ecstasy* and *grief*). Its revised version [18] improves consistency, removed neutral emotions (e.g., *surprise* was eliminated as annotators found it difficult to decide its valence), and adds polar and self-conscious emotions.

A recent survey [19] examines most of the sentiment analysis surveys published during the last decade and compiles a list of the most promising research directions. The number of papers has increased massively during the past 20 years (from two papers in 2002 to 1466 in 2021). The survey identifies six large communities (social media, ML, NLP, opinion mining, Arabic, semi-supervised learning) and their key research topics.

Domain-specific affective models [20] go beyond classic models by incorporating application-specific affective categories and interpreting them based on the situational context. An affective model for benchmarking TV shows, for example, might consider *fear* and *sadness* to be desirable associations rather than undesirable ones.

### Lexicon-Based and Bag-of-Words Models for Financial Sentiment Analysis

The use of sentiment as an indicator to support decision-making in finance has a long history; however, early proprietary systems have been rarely documented in academic literature.

Sezer’s work [21] provides a systematic review of deep learning models for financial time series forecasting used for stock and commodity prices during the late pre-COVID era (2005–2019). The survey reviews the mathematics behind each model and its parameters, as well as the markets to which it was applied. Recurrent neural network models (RNN) like long short-term memory networks (LSTM) or gated recurrent units (GRU) are most common, followed by convolutional neural networks (CNN), regardless of the market.

Due to their generalization capabilities, LSTM models are also well-suited for commodity prices and can be used for predicting prices of WTI or Brent crude oil daily closings [22]. An LSTM-DNN hybrid model trained on multiple markets, including oil, yielded the best results for predicting coal prices [23]. When considering these results, it is important to note that most of the presented methods have been evaluated on different datasets and time intervals, which seriously impacts the comparability of results.

One of the most cited studies addressing the use of NLP and lexicons for FSA is Loughran and McDonald’s work on interpreting liabilities associated with 10-K filing returns [24]. They created a large corpus of 10-K samples from the Electronic Data Gathering, Analysis, and Retrieval (EDGAR) website.^2^ Their analysis focuses on the management discussion & analysis (MD&A) sections to examine the most frequent words and negative terms included in financial dictionaries that are found in the fillings. The study concludes that some words with negative connotations may be industry-specific and not indicative of any liabilities; therefore, general categorization schemes need to be used with caution. The same authors have also published a longer survey on the use of text analysis in accounting and finance [25]. This survey covers mostly the analysis of public financial documents like US Securities and Exchange Commission (SEC) fillings over three decades of text analytics (1984–2014) and reviews financial lexicons, bag-of-words methods, and document similarity metrics.

Xing et al. [26] present a review of surveys and a taxonomy of early NLP models for natural language-based financial forecasting (NLFF) covering the pre-Transformer era. They focus mainly on literature that aims at predicting the stock market using bag-of-words approaches, as well as early ML methods (e.g., k-NN, SVM, least squares regression, and decision trees). The article considers NLFF a nascent field and defines its areas of interest (e.g., sentiment, volatility, technical and fundamental analysis, portfolio management). It also goes on to describe the most important models (e.g., GARCH, ARIMA, neural networks), and methods (e.g., simulation, credit scoring, exchange rates, backtesting) used in this field.

Sentiment-based forecasting methods for crude oil prices are discussed in [27]. The article presents a method that uses a CNN to extract sentiment features. They suggest that text features and financial (e.g., numerical) features are somewhat complementary.

### Deep Learning Models for Financial Sentiment Analysis

A recent survey on deep learning for text classification [28] considers sentiment analysis a crucial text classification task. The authors group sentiment analysis methods into two classes (rule-based and ML-based) and define a set of classification tasks (e.g., sentiment analysis, news classification, topic analysis, question answering, textual entailment). The survey is particularly interesting since it provides guidelines and steps (model selection, domain adaptation, task-specific model design, task-specific fine-tuning, and model compression) for selecting and adapting neural networks to a particular task. In addition, it also includes information on resources and evaluation metrics.

FSA is not a new field as sentiment indicators have already been used as early as in the 1980s, although most early systems were proprietary and, therefore, not properly described in the literature. A recent analysis of the common errors and successful approaches in FSA was provided by Xing et al. [29]. The article compares eight models for the FSA task on the Yelp and StockSen datasets and provides a list of six common errors. These include rhetoric issues, dependent opinions, counterfactual moods (irrealis), unspecified aspects (e.g., cases in which humans discover the correct aspect easier than the algorithms), unrecognized words (e.g., acronyms), and external references (e.g., references that need additional information).

The public mood has been a reliable indicator of market direction, but until recently, it has rarely been used for intelligent asset allocation [30]. Work by Xing et al. shows that market views, a formalization of the public mood, can help increase returns when combined with Bayesian allocation models. Their experiments also prove that such an approach increases profitability by 5 to 10% annually. In subsequent work [31], they draw upon LSTMs and adapted their approach to automatic portfolio management, raising annual profitability by 19%.

The sentiment itself can be used for estimating various target metrics, which makes it difficult to assess its predictive power. A survey on sentiment analysis based on deep learning [32] enumerates several widely used models from CNNs and RNNs to LSTMs, GRUs, and hybrid networks, and provides a taxonomy of sentiment analysis techniques. The surveyed methods include ML approaches (e.g., semi-supervised learning, unsupervised and supervised), lexicon-based techniques (e.g., dictionary-based, corpus-based), and hybrid methods (e.g., combinations of neural networks and lexicons or corpus). Li et al. [33] draw upon sentiment for predicting prices and presents an LSTM model that considers technical indicators and sentiment analysis. Xing et al. [34] use sentiment for predicting volatility and implements a system called SAVING (sentiment-aware volatility forecasting) which is built upon a variational RNN (VRNN).

### Foundation Models for Financial Sentiment Analysis

The first Transformer model was published in 2017 [35], but due to its flexibility, this class of models has been used for solving many problems in areas such as NLP and computer vision.

Financial Transformer models typically use encoder pre-training on broad collections of data. These models are suitable for a wide number of downstream tasks and can also infer logic statements. Due to their central role in the NLP ecosystem, and their incomplete nature (i.e., they require fine-tuning since logical statements are inferred based on the training data only), these Transformer models are called foundation models [36]. Such models are enabled by transfer learning and can scale to large volumes of data.

Most current work on FSA is based on the architectures of the Bidirectional Encoder Representations from Transformers (BERT) transformer [37], specifically versions of the FinBERT model [38]. FinBERT was one of the first language models applied to the finance domain. When trained on the FiQA dataset^3^ and compared with ELMo and ULMFit, it achieved an improvement of 15% in accuracy. The works from Yi et al. [39] further improve FinBERT’s performance by training on multiple datasets (e.g., Financial Phrase Bank, AnalysTone, and FiQA) and optimizing fine-tuning strategies. Liu et al. [40] train FinBERT on five large general-purpose datasets and on six self-supervised tasks (dialogue relation, sentence distance, reshuffling, token-passage, capitalization, and span prediction) to create a more robust FinBERT version. Their model outperforms other approaches for all evaluation tasks described in their paper (e.g., Financial Sentence Boundary Detection, FSA, and Financial Question Answering). FinBERT can be used in a multitude of applications (e.g., FinTextSen, FinNum tasks, sentiment analysis, numeral understanding) and domain adaptation seems to help improve results, although various errors suggest that the fine-tuning process on small datasets is not always stable [41]. Researchers also use FinBERT as a feature extractor for financial text classification [42] and prediction.

A recent article analyzes FinBERT’s sentiment performance, as well as its advantages over classic BERT large language models (LLMs) [43]. FLANG models [44] expand upon FinBERT and benefit from training on a benchmark known as the Financial Language Understanding Evaluation (FLUE) benchmark.

Zou and Herremans [45] combine FinBERT embeddings with a multimodal model to predict extreme price fluctuations of cryptocurrencies in Twitter feeds. Each year, the best Transformer-based financial models compete in various workshops on classification, phrase similarity, or sentiment-related tasks. During the last few years, the top systems were based on FinBERT [46].

Compared to the number of papers that used FinBERT as a backend for classification, the amount of literature that builds upon its capabilities as a feature extractor is relatively small. Farimani et al. [47] use a FinBERT model fine-tuned for FSA as a backend for an RNN feature extractor and implements an API for cryptocurrency markets. The API as well as their BERT model (FinBERT-SIMF) is publicly available. Ider [48] trains a BERT-based sentiment model on Reddit and Twitter posts for predicting market movement. The model lacks the capability to correctly assign sentiment if multiple targets are involved (e.g., if one target is negative, both targets may end up labeled negative). Chuang and Yang [49] analyze the output of BERT and FinBERT models, and discover that they have some positive implicit preferences towards the stock market and some serious differences in the treatment of various industries.

Closer to the domain of our article, Fang et al. [50] showcase a hybrid model for crude oil price forecasting that integrates FinBERT, variational mode decomposition (VMD), attention mechanisms, and a BiGRU DL model. VMD is a modern signal decomposition technique that was recently used by Huang and Deng [51] for modeling crude oil prices. Since VMD is typically used in hybrid models, its role is that of a time series cleaning method, and it is generally coupled with LSTMs and GRU models. A language model focused on sentiment for the oil and gas domain called PetroBERT [52] was also identified, but only available in Portuguese, which makes comparative evaluations with English models difficult.

The massive amount of recent literature on FSA demonstrates its importance as a field. Nevertheless, much research focuses on incremental improvements of existing models, and rarely considers economic theory, although market behavior should ultimately adhere to fundamental economic laws. The work presented in this article addresses this issue by blending existing affective models with economic theory.

## Problem Statement

To gain insights into the impact of media coverage on crude oil markets, we investigated the accuracy of cumulative sentiment scores returned by FinBERT based on news media headlines. Initial experiments revealed a surprisingly poor relationship between the aggregated sentiment classification results and WTI crude oil futures prices (Fig. 7).

An investigation that analyzed FinBERT scores for a random sample of new headlines revealed systematic problems in FinBERT’s capability to distinguish between an event’s sentiment and its impact on the oil price.

Table 1 provides a number of real-world examples that illustrate these issues. For example, headlines reporting accidents at oil production facilities usually indicate a drop in supply, leading to higher crude oil prices. However, the FinBERT model tends to classify such events as negative, since they are usually not seen as good news, and typically lead to negative consequences such as disruptions of business operations and financial losses.

On the other hand, headlines implying a rise in supply (e.g., discoveries, higher production, and higher exports) usually lead to lower prices. FinBERT, in contrast, frequently classifies such news as neutral. Decreasing demand (e.g., due to lower imports) typically leads to lower prices. Another observed limitation is that FinBERT correctly captures headlines that explicitly mention keywords such as “down,” “lower,” or “decreases,” but fails to do so if the decrease is implied by negative numbers (e.g., “−16.0%”).

**Table 1:**
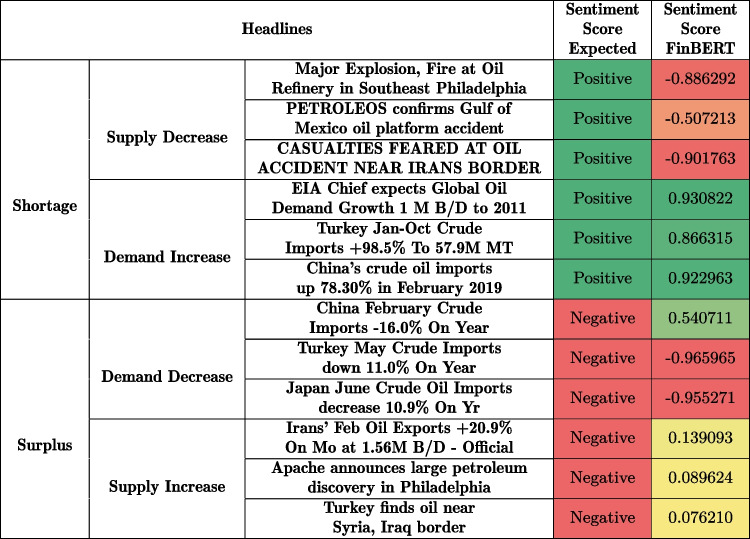
Expected and actual FinBERT sentiment scores

Pos. = green, Neu. = yellow, Neg. = red

Some of the behavior described above is typically expected and considered a domain adaptation problem that arises from using general sentiment analysis methods in a new domain [29]. However, FinBERT was specifically trained on financial news [38]. Araci [38] performed its domain adaptation process by training on a subset of the Thomson Reuters Text Research Collection (TRC2) where occurrences of slang and spelling errors are minimal. For the task-specific fine-tuning process, the training dataset Financial Phrase Bank from [53] was utilized.

**Fig. 1:**
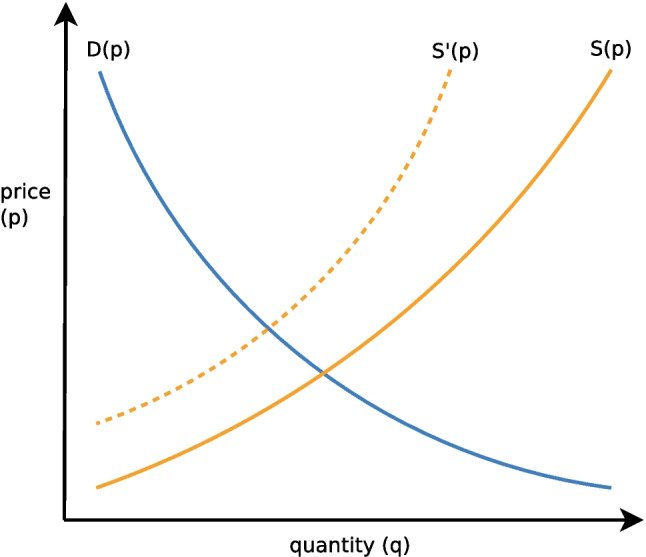
Adam Smith’s law of supply and demand. The dashed supply $S'(p)$ curve shows the impact of a supply shortage on the equilibrium price and quantities

The examples collected in Table 1 illustrate that FinBERT could clearly benefit from economic theory. Adam Smith’s law of supply and demand [54] seems particularly well suited for determining the impact of market events on future commodity prices. The law states that the price of a good is determined by the interplay of its supply and demand. Supply refers to the amount of a product or service available in the market, while demand is defined as the quantity buyers are willing to acquire. Both supply and demand depend on the market price (Fig. 1) since producers will increase supply at higher prices and buyers will purchase higher quantities if prices are low.

In a perfect market, the intersection of the supply (*S*(*p*)) and demand curves (*D*(*p*)) determines the equilibrium price and quantity. The equilibrium price is the price at which goods are traded. The corresponding equilibrium quantity refers to the amount of goods that producers are prepared to sell at that price and also the number of goods that buyers are willing to purchase.

Equipping affective models with this knowledge allows them to reasonably interpret market events. For example, if demand remains constant, but supply falls (e.g., due to an event with a negative sentiment such as an accident), the supply curve will shift to the left ($S'(p)$; see Fig. 1) and the shortage will cause a higher equilibrium price. A shortage might also occur if the supply remains constant, but demand rises (e.g., due to a positive market event such as high economic growth). Increased supply with constant demand, on the other hand, would result in a surplus and, as a consequence, lower prices. A surplus also occurs if supply remains constant while demand falls. The logic behind supply and demand can be summed up as follows [54]:

- Less supply $\rightarrow$ shortage $\rightarrow$ higher price
- More supply $\rightarrow$ surplus $\rightarrow$ lower price
- Less demand $\rightarrow$ surplus $\rightarrow$ lower price
- More demand $\rightarrow$ shortage $\rightarrow$ higher price

The economic theory of supply and demand considerably improves the understanding of market dynamics [2]. Nevertheless, it may not always be sufficient to explain market behavior, since regulatory laws, cartels, automated bots, and other mechanisms might bias or disrupt markets. In addition, new unpredictable events such as the recent coronavirus pandemic might cause market uncertainty, particularly concerning future supply, demand, and price movement.

Consequently, economic models only cover a fraction of all possible scenarios and require adaptation to the industry and market in which they are expected to operate. Nevertheless, they can considerably improve the performance of domain-specific affective models since they help in better interpreting market events.

The EMH is another economic model of interest, which postulates that financial markets are informationally efficient and that current prices reflect all publicly available information, making it impossible to consistently achieve abnormal returns through the use of technical or fundamental analysis [1]. However, there are articles suggesting that the crude oil market by itself is not efficient [55]. Shambora and Rossiter [55], for instance, show that complex buy-and-hold strategies coupled with technical analysis can exploit market inefficiencies. Using additional indicators for predicting likely market movements could help in capitalizing on such inefficiencies. Sentiment in and of itself, for example, provides clues on future market movements and can be enriched with additional indicators.

This paper aims at identifying and applying such indicators to existing language models to create systems that provide reasonable predictions of the direction and amplitude of price movements in the commodity markets. WTI crude oil futures act as a test bed for this study, since they are homogeneous in nature and usually less volatile than other commodities such as coffee, wheat, and corn.

## Method

The following section discusses the design of CrudeBERT+, a domain-specific affective model that aims at predicting changes in crude oil prices. The method presented here is hybrid, as it combines language models with sentiment analysis, background knowledge on entities, numerical clues, and social knowledge (e.g., Google Trends).

Figure 4 provides a short overview of the proposed method. Fine-tuning FinBERT with domain-specific affective knowledge yields the CrudeBERT classifier (“CrudeBERT” section) [56]. Further enrichment of CrudeBERT with common knowledge (NE), numeric clues (NC), and Google Trends (GT) that is processed using heuristic reasoning yields the CrudeBERT+ model (“CrudeBERT+” section):

1. The CrudeBERT model estimates the economic effect of news and aims at predicting their impact on crude oil prices. The base model is particularly well-suited for determining the *direction* of a price change since it has been fine-tuned with data that illustrates Adam Smith’s law of supply and demand.
2. The proposed refinements aim at collecting information used for estimating the *strength* of the price movement.

### Dataset

A dataset comprising 26,270 headlines from 1 January 2012 to 1 April 2021 has been obtained through the *RavenPack Realtime News Discovery*^4^ platform by querying for headlines labeled as “highly relevant” towards RavenPack’s “crude oil” category. The headlines originate from 1034 unique news sources, including *Dow Jones Newswires* (6609), *Reuters* (2095), *Bloomberg* News (933), and *Platts* (768). The dataset also considers 329 sources that only delivered a single headline. The *WTI crude oil futures* prices for the same period (January 2012–April 2021) were acquired from Investing.com*.*^5^

It is important to note that commodities have generally two agreed prices: (i) spot — which reflects the price upon the purchase of the commodity with delivery on the same day, and (ii) futures — which refers to the price of a contract with delivery at a future date. Futures contracts, therefore, also reflect the cost of storing and in some cases transporting the commodity during the respective period. In this article, we only consider future contracts, as they are more likely to indicate the impact of current events on the prices.

### CrudeBERT

CrudeBERT is a variant of FinBERT [38] that has been fine-tuned towards assessing the impact of market events on the WTI crude oil future prices. The fine-tuning dataset focuses on frequently occurring market events, and their impact on market prices according to Adam Smith’s theory of supply and demand. To create a suitable silver standard dataset, we collected frequently recurring topics from the domain of crude oil markets in an ontology. These topics, the corresponding trigger terms, and their polarities have been used as background knowledge for interpreting the polarity of a headline’s sentiment based on supply and demand. Table 2 contains an excerpt of terms and synonyms used within the system.

**Table 2:** Excerpt of terms and synonyms used in the crude oil markets domain ontology

**Example terms used in query search to detect polarities**				
**INCREASE**	**NO CHANGE**	**DECREASE**	**PIPELINE**	**ACCIDENT**
“+”	FLAT	“-”	BLOW	ACCIDENT
BOOST	FREEZE	BEARISH	BOMB	CASUALTIES
BULLISH	HOLD	BOTTOM	CONSTRAINED	CRASH
UP	SAME	DOWN	EXPLODE	DEAD
INCREASE	STEADY	SINKING	LEAKS	DEATH
...	...	...	...	...

The ontology contains (i) topics with metadata on the change’s direction (e.g., rising oil prices, changes in import and export volumes, oil supply and demand), and (ii) topics without directional data, such as oil spills, accidents, pipeline constraints, oil discoveries, and drilling (Table 3). The fine-tuning process relies upon a five-fold cross-validation process that uses 80% of the available silver standard dataset. The remaining 20% are used for testing, i.e., to obtain an unbiased estimation of the model’s classification performance on unseen data.

**Table 3:** Frequency of reoccurring topics in the crude oil domain

**Supply change**			**Demand change**		
Increase	No change	Decrease	Increase	No change	Decrease
ca. 5900	ca. 350	ca. 5700	ca. 1300	ca. 50	ca. 800
**Export change**			**Import change**		
Increase	No change	Decrease	Increase	No change	Decrease
ca. 2000	ca. 150	ca. 1500	ca. 2800	ca. 50	ca. 2300
**Price change**			**Spill**	**Discovery**	**Drilling**
Increase	Decrease		ca. 2300	ca. 1600	ca. 100
ca. 1600	ca. 1300		**Accident**	**Pipeline issue**	
			ca. 400	ca. 100	

**Fig. 2:**
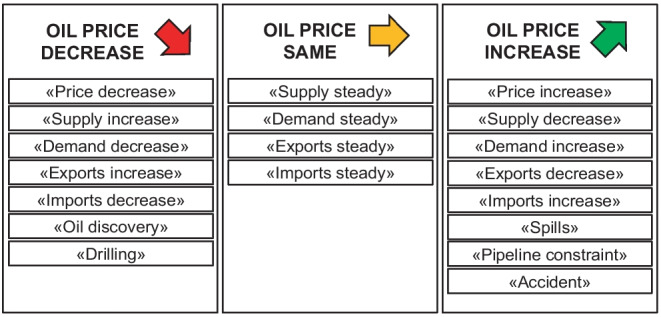
Frequently occurring topics and their impact on the oil price

The system then labels matches between these topics and news headlines and uses them to predict their impact on crude oil prices, as illustrated in Fig. 2.

**Fig. 3:**
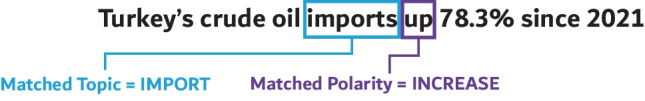
Identifying topics and direction in headlines

The following headline (Fig. 3), for instance, indicates a higher demand due to an *import increase*. The model, therefore, assumes that the oil price should rise.

### CrudeBERT+

**Fig. 4:**
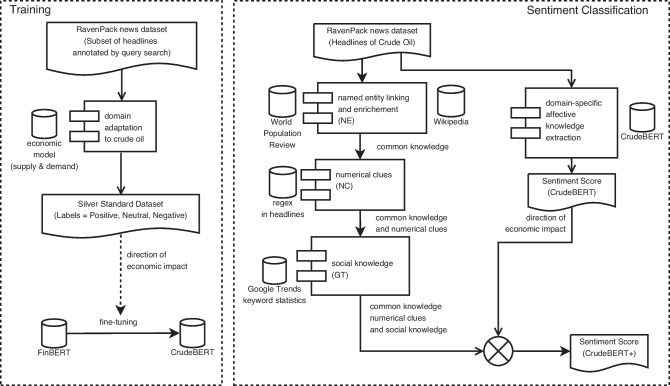
Overview of the CrudeBERT and CrudeBERT+ affective models

**Fig. 5:**
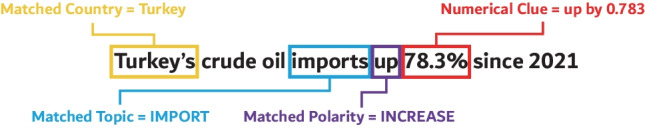
Identifying common knowledge and numerical clues in headlines

As outlined in Fig. 5, CrudeBERT+ improves upon CrudeBERT by considering background knowledge that has been extracted by a common knowledge extraction pipeline which identifies mentions of companies, and countries within news article headlines. For this purpose, CrudeBERT+ draws upon background knowledge mined from structured knowledge repositories such as *World Population Review*^6^ and *Wikipedia*^7^ to enrich these entities with common knowledge that is used to assess the headline’s impact on the overall oil price. In addition, numeric values (e.g., absolute numbers, percentages) and social knowledge derived from *Google Trends*^8^ are extracted to further aid in quantifying these effects (Fig. 4).

The headline “*Iraq*’s Feb Oil Exports *+20.9%* On Mo At 1.56 M B/D,” for instance, mentions the country *Iraq* (GDP volume: 160B USD/rank: 49; population: 42.2M, oil export volume: 4.6B barrel per day/rank: 4; oil consumption volume: 0.85B barrels per day/25) and the percentage value *20.9%* which allows quantifying the event’s effect in relation to the country’s individual impact. In addition, CrudeBERT+ is aware that the headline refers to oil exports.

#### Integration of Common Knowledge on Named Entities (NE)

We draw upon named entity linking (NEL) for identifying companies and countries within the article headlines. As discussed above, these entities are then enriched with indicators that help in assessing the headline’s relative importance and impact. The current version of CrudeBERT+ considers the following indicators, although additional ones might be added in future versions:

- Oil companies: share of revenue in relation to the total revenue earned by the world’s top-46 oil companies and their revenue rank among these companies.
- Countries: share in the world’s gross domestic product (GDP) as well as their rank in terms of GDP

Extensive experiments and drill-down analyses yielded the presented heuristic approach for estimating an entity’s impact on market prices. We use the named entities mentioned in the news headlines to compute discount factors $S_{company} \in [0, 1]$ (Eq. 3) and $S_{country} \in [0, 1]$ (Eq. 6).

CrudeBERT+ assesses the impact of the mentioned **oil companies** $o\in O$ based on their total market share in terms of revenue ($rev(o)/rev_{total}$) and their relative revenue rank ($r_{rev}(o)$). $O_{all}$ refers to the oil companies available in our database (i.e., the top-46 oil companies listed in Wikipedia). The minimum impact values $S_{min} = 0.1$ ensures that even headlines covering smaller companies maintain a certain influence.

1 $$\begin{aligned} S(o)&= \max \left( \frac{rev(o)}{rev_{total}} + \frac{1}{\sqrt{r_{rev}(o) + 1}}, S_{min}\right) \end{aligned}$$

2 $$\begin{aligned} rev_{total}&= \sum _{o\, \in\, O_{all}} rev(o) \end{aligned}$$

3 $$\begin{aligned} S_{company}&= {\left\{ \begin{array}{ll} 1.0 &{} \text {if } O = \emptyset \\ S(o) &{} \text {otherwise.} \end{array}\right. } \end{aligned}$$

The system does not discount the importance of headlines that do not mention any oil companies (i.e., $O = \emptyset$) and, therefore, yields an $S_{company}$ of 1.0 in such cases.

We assess each **country** ($c \in C$) that has been identified within the headline based on its relative share of the world’s GDP ($gdp(c)/gdp_{total}$) and its GDP rank ($r_{gdp}(c)$).

4 $$\begin{aligned} S(c)= & {} \max \left( \frac{gdp(c)}{gdp_{world}} + \frac{1}{\sqrt{r_{gdp}(c) + 1}}, S_{min}\right) \end{aligned}$$

5 $$\begin{aligned} gdp_{world}= & {} \sum _{c\, \in\, C_{all}} gdp(c) \end{aligned}$$

6 $$\begin{aligned} S_{country}= & {} {\left\{ \begin{array}{ll} 1.0 &{} \text {if } C = \emptyset \\ \min _{c\, \in\, C} S(c) &{} \text {otherwise.} \end{array}\right. } \end{aligned}$$

News coverage on trade relations frequently leads to mentions of multiple countries. In these cases, we only consider the lowest impact score (Eq. 6), since the smaller partner usually determines the total size of a trade. If no countries are mentioned in a headline (i.e., *C* = $\emptyset$), it is assigned a factor of 1.0, i.e., no discounting takes place.

Finally, we combine both discount factors as outlined in Eq. 7 to obtain an estimation of the headline’s total impact.

7 $$\begin{aligned} S_{NE}= & {} S_{company} \cdot S_{country} \end{aligned}$$

#### Integration of Numerical Clues (NC)

As illustrated in Fig. 5, CrudeBERT+ identifies mentions of percentages ($p \in P$) in headlines. The factor $S_{NC}$ indicates the perceived impact of the headline based on these numerical clues.

8 $$\begin{aligned} S_{NC}&= {\left\{ \begin{array}{ll} 1.0 &{} \text {if } P = \emptyset \\ \frac{min_{p\, \in\, P}(p)}{100} &{} \text {otherwise.} \end{array}\right. } \end{aligned}$$

If multiple clues occur, the limiting factor (i.e., the smallest percentage value) is used.

#### Integration of Social Knowledge of Google Trends (GT)

CrudeBERT+ also considers social knowledge by observing the daily web search statistics reported on *Google Trends*^9^ for *crude oil* and related topics such as petroleum, gasoline, and diesel. These statistics provide an indicator $GT_{oil} \in [0, 100]$ that correlates with the number of actors involved and interested in current events in the crude oil market. Refining the aggregated sentiment scores of CrudeBERT with the factor $S_{GT}$ (Eq. 9) indicates the headline’s importance in terms of the Google Trends search statistics. The incorporation of social knowledge in our analysis bears resemblance to the examination of daily news volume, as it has the potential to provide valuable insights into the possible market effect of an event. This notion is supported by previous research from Hafez et al. [57], which suggests that the volume of news coverage is positively correlated with market activity. In their experiments, they emphasize the importance of news volume and quote better results as reasons for accumulating daily sentiment scores rather than computing daily averages.

9 $$\begin{aligned} S_{GT} = {\left\{ \begin{array}{ll} S_{GT_{min}} &{} \text {if } GT_{oil} < S_{GT_{min}} \\ GT_{oil} &{} \text {otherwise.} \end{array}\right. } \end{aligned}$$

The minimum Google Trends impact value $S_{GT_{min}} = 1.0$ reflects that even headlines published during periods of low media coverage do have an impact on the WTI crude oil future price.

## Evaluation

### Benchmark Dataset

The benchmark dataset consists of RavenPack headlines, and information on their publishing date and source, which have been retrieved according to the information provided in the “Dataset” section. The dataset also contains annotations that classify headlines according to the following affective models: (i) a proprietary *Event Sentiment Score* provided by RavenPack, (ii) the sentiment computed by FinBERT, and (iii) market sentiment scores computed by CrudeBERT (“CrudeBERT” section) and CrudeBERT+ (“CrudeBERT+” section). In addition, a column with the corresponding WTI crude oil futures prices is provided.

The dataset is available on GitHub,^10^ although we had to replace the headlines with their SHA-256 hashes for copyrights reasons. The full dataset can be recreated by downloading the headlines from RavenPack as outlined in the “Dataset” section and then calling the mapping script provided in the repository.

### Preprocessing

We normalize all price ($price_t$) and sentiment ($sent_t$) values using min-/max scaling as outlined in Eqs. 10 and 11:

10 $$\begin{aligned} sent^{norm}_t = \frac{sent_{t}-sent_{min}}{sent_{max}-sent_{min}} \end{aligned}$$

11 $$\begin{aligned} price^{norm}_t =&\frac{price_{t}-price_{min}}{price_{max}-price_{min}} \end{aligned}$$

where $sent_{max}$, $price_{max}$ refers to the maximum sentiment and price values within the dataset, and $sent_{min}$, $price_{min}$ to the corresponding minimum values.

**Fig. 6:**
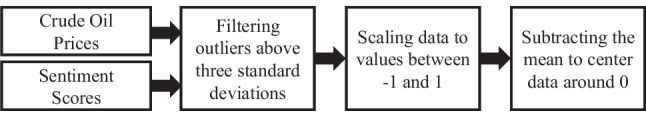
CrudeBERT’s data preprocessing pipeline

The sentiment data normalization aims at integrating the market’s relative mood into the CrudeBERT+ prediction model, and price data normalization helps in better coping with extreme market movements. In addition, a filtering step removed outliers by filtering columns with changes that exceeded three standard deviations (Fig. 6). The preprocessing deleted a single data point out of 2417. On 20 April 2020, the WTI futures experienced a drastic devaluation and ended up in an unprecedented negative pricing, which has been removed from the dataset.

### Evaluation Metrics

**Fig. 7:**
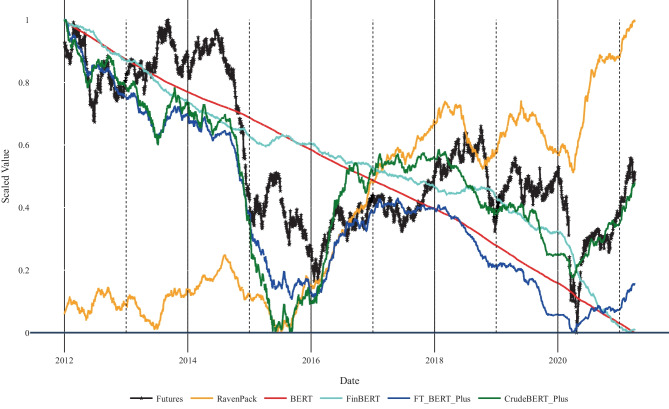
Comparison of WTI crude oil futures prices with oil prices movements predicted based on news headlines published between January 2012 and April 2021. The graph illustrates the models’ capabilities in tracking real-world prices based on the news headlines dataset

The domain-specific affective models introduced in this paper analyze news media headlines to generate predictions for WTI crude oil futures prices. The evaluation, therefore, aims at measuring the degree of association between the model’s predictions (i.e., the cumulative sentiment scores in Fig. 7) and the movements of WTI oil price futures.

Consequently, it is important to identify performance metrics that truly capture this objective. Correlation in its different forms (e.g., Pearson, Spearman, Kendall) generally measures the strength of an association, as well as its direction. An early article that compares pattern analysis algorithms suggested that regional correlation, dynamic time warping (DTW), and skeletal tree matching can all produce similar results [58]. However, a good correlation score does not necessarily translate into a good approximation of time series data, since regression frequently yields better scores than curves that more accurately track the target time series. We, therefore, aimed to use a metric that also successfully captures the visual intuition behind approximating curves, namely that the distance between the predicted curve and the target curve should be small.

An entire class of such distances exists: Needleman-Wunsch, Smith-Waterman, Levenshtein, and DTW [59]. They are all metrics that belong to a class of sequence alignment algorithms. For time series, especially in contexts of semantic multimedia (e.g., speech or video processing), DTW was one of the most used distances. DTW is a cost metric based on the Levenshtein distance; therefore, it can also intuitively convey the fact that lower scores signify a better fit between the predicted and the gold standard curve. The lower the scores, the better.

A recent article by Linke et al. [60] shows that in the field of neuroimaging, DTW outperforms Pearson’s correlation in detecting certain spectrum disorders. They explain that DTW is more robust to lag, test-retest reliability, and task-related changes. Vaughan et al. [61] provide a comprehensive analysis of how to apply DTW to time series analysis. They outline its use for comparing different time series and discuss methods for removing sections from time series, compressing and combining them. Closer to our application domain, Bai and his team [62] showcase the application of DTW to financial time series analysis, particularly for tasks such as identifying the most dominant series and entropy values.

DTW computes a minimal distance between trajectories using a cost function. Given two time series $ts_{1}$ and $ts_{2}$ with the associate measurement sets P and S, DTW constructs a matrix with P$\times$S cells. The cells are colored black and white based on the difference between the two insertions; black indicates large differences, whereas white indicates a lack of differences. The path is then constructed through the white zones, avoiding the black areas if possible. The resulting minimal path is colored red. A distance measure $\sigma$ is then used to compute the distances between each series and the minimal path, for example:

12 $$\begin{aligned} \sigma (i,k)= |ts_{i}-ts_{k}| \end{aligned}$$

13 $$\begin{aligned} \sigma (i,k)= (ts_{i}-ts_{k})^2 \end{aligned}$$

DTW can be expressed as the minimal distance over the two potential paths between time series as (based on [61]):

14 $$\begin{aligned} DTW(ts_{i},ts_{k})= min[\Sigma _{k\,=\,0}^p\sigma (w_{s})] \end{aligned}$$

### Evaluation Task

*The evaluation task* predicts the impact of events mentioned in news headlines on crude oil prices. We then use DTW to compare the cumulative computed prices (i.e., domain-specific sentiment scores) with the ones obtained from the WTI crude oil futures over the investigated time period. The evaluation considers the performance of predictions within particular years (i.e., the yearly columns in Table 4), and the continuous DTW distances between oil prices and the cumulative sentiment (i.e., the performance of prediction over the whole time period from January 2012 to April 2021). The year 2021 is not included in the yearly analysis, since the dataset only covers the first 4 months of that year.

### Results

The introduced domain-specific affective model aims at calculating a proxy metric that closely approximates the selected target measure in the domain of crude oil prices. The evaluation setting considered two different kinds of target metrics: (i) the crude oil price that is discussed in Table 4, and (ii) changes to the crude oil price which refers to the first derivative of the price. Eventually, since the results were similar, we settled on the price only.

Using sentiment as a proxy for predicting crude oil prices assumes that the market mood should be reflected in the current price, with minimal adoption of expectations due to an existing price level. The alternative hypothesis assumes that market mood quickly adapts to a new price level (i.e., the new normal) and that positive or negative sentiment value should trigger corresponding changes (e.g., higher or lower prices) in the market.

The results in Table 4 indicate that the sentiment scores of the CrudeBERT+ variants have lower DTW distance, thus, better approximate the price than other sentiment classifiers. The DTW results for the entire dataset (i.e., 2012–2021; last column of Table 4) correspond to the curves in Fig. 7. CrudeBERT+ helped to reduce the DTW by a factor of 8.1 when compared to RavenPack, a factor of 2.2 compared to BERT, and 2.1 when compared to FinBERT. These results indicate that the suggested approach significantly improves the predictive power of BERT and FinBERT models. The DTW analysis also reveals that incorporating *Google Trends* keyword statistics triggers a further reduction of the DTW distance. This observation is also valid, for most of the shorter yearly evaluation periods.

**Table 4:**
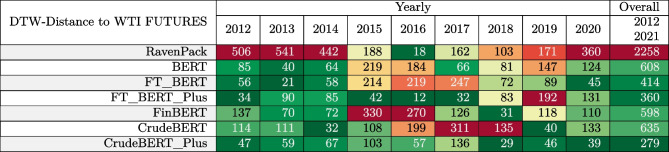
DTW distance between WTI crude oil future prices and cumulative sentiment scores

Colors indicate the fit between the model’s prediction and the WTI crude oil future price (green = good, yellow = reasonable, red = poor)

**Table 5:**
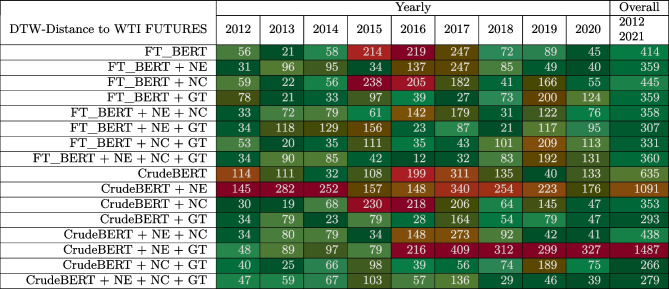
Ablation study comparing the impact of different feature sets on the CrudeBERT and FT_BERT models

Colors indicate the fit between the model’s predication and the WTI crude oil future price (green = good, yellow = reasonable, red = poor)

**Table 6:** Sentiment classification performance on the silver standard dataset

Metric	Category	BERT	FT_BERT	FinBERT	CrudeBERT
Precision	Positive	0.52	**0.98**	0.40	**0.98**
	Neutral	0.00	**0.99**	0.02	0.96
	Negative	0.34	**0.97**	0.33	**0.97**
	Macro	0.29	**0.98**	0.25	0.97
Recall	Positive	**0.99**	0.97	0.28	0.97
	Neutral	0.00	0.96	0.25	0.96
	Negative	0.00	0.97	0.31	**0.98**
	Macro	0.33	**0.97**	0.28	**0.97**
F1-score	Positive	0.68	**0.98**	0.33	**0.98**
	Neutral	0.00	**0.98**	0.04	0.97
	Negative	0.01	**0.97**	0.32	0.96
	Macro	0.23	**0.97**	0.23	**0.97**

Values in bold indicate best results

**Fig. 8:**
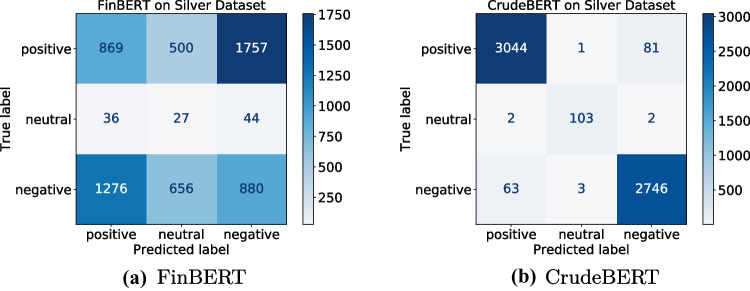
Confusion matrices for FinBERT and CrudeBERT on the silver standard dataset

## Discussion

This paper introduces several variants of CrudeBERT, domain-specific affective models optimized for predicting crude oil markets. The models improve upon BERT and FinBERT by leveraging economic theory and background knowledge on entities such as countries and companies that aid in interpreting the sentiment expressed in news headlines.

When we started work at CrudeBERT, we were expecting marginal improvements upon the original FinBERT model. Our early observation that FinBERT often fails to detect trend changes properly (“Problem Statement” section) triggered a thorough drill-down analysis which revealed the model’s lack of contextual knowledge required for correctly interpreting a wide range of market events. Further investigations revealed that BERT handles neutral news headlines better than FinBERT, since BERT was trained on a neutral corpus [49]. The overall positivity of the human language provided another implicit bias [63]. In addition, domain-specific language like the one used for trading can also be difficult to interpret (e.g., a statement about the market going up, can still end up being negative depending on the context, even if it does not contain any negative words) [24]. Given the amount and significance of more nuanced market events, domain adaptation without a sufficient amount of neutral examples leads to serious shortcomings in the trained models.

Figure 8 illustrates that even a classic BERT model that lacks any domain adaptation will perform well with the fine-tuning processes used for CrudeBERT. We, therefore, also included the BERT_FT models in our analysis. Considering economic theories, particularly the law of supply and demand, is another key ingredient in addressing the mentioned shortcomings. Improving CrudeBERT to CrudeBERT+ led to the integration of common knowledge, numerical clues, and social knowledge into the model, as they provide additional insights that help in evaluating an event’s scope and impact.

As indicated by the lower DTW distances in Tables 4 and 5, CrudeBERT and CrudeBERT+ outperformed the other models over the whole 9-year period as well as in most of the investigated individual yearly slices. These results are particularly interesting when considering the vast macroeconomic differences between the observed time intervals, which even included the start of the 2020 pandemic. The COVID pandemic led to rare market conditions such as correlations between multiple markets [11], and even to previously unseen conditions such as negative oil prices. The good performance of CrudeBERT variants in these settings is somewhat surprising and deserves further examination.

Table 6 compares the sentiment classification performance of the initial models (BERT, FinBERT) to their fine-tuned counterparts (FT_BERT, CrudeBERT). These results clearly indicate that the fine-tuning process used for creating CrudeBERT benefits general language models (e.g., BERT), as well as models that have already been adapted to a specific domain (e.g., FinBERT). Since CrudeBERT and CrudeBERT+ perform similar for the sentiment classification tasks, we only report CrudeBERT’s scores.

Table 5 summarizes the results of an ablation study on the fine-tuned BERT (FT_BERT) and CrudeBERT models that considers all relevant feature combinations (i.e., use of common knowledge on named entities (NE), numerical clues (NC), and Google Trends (GT)). The ablation study shows that common knowledge on entities (NE) provides a larger benefit to FT_BERT than to CrudeBERT. The other two feature sets (numerical clues and Google Trends) do have similar effects on both models. Adding Google Trends as a feature seems to provide a balancing effect if numerical clues are included as well. Considering all three feature sets yields the best performance for both models, as expected. These results suggest that adding common knowledge, numerical clues, and features derived from Google Trends works well for BERT, FinBERT, and CrudeBERT (which was built on top of FinBERT).

The introduced method applies CrudeBERT and its variants to headlines rather than full news articles. Even though the articles themselves contain more detailed information, relying solely on headlines has considerable advantages in terms of pre-processing, data storage, and computational power. In addition, using headlines significantly limits noise in the form of stop words and negates. Work by [64] and [27] also draws upon headlines, considering them as summaries of the full articles, which are more convenient to process.

## Outlook and Conclusion

CrudeBERT and its variants (i) leverage economic theory, (ii) draw upon common knowledge to derive features based on the named entities mentioned in news headlines, (iii) collect numerical indicators, and (iv) consider data derived from Google Trends to better interpret market events. As confirmed in the evaluations, the domain-specific knowledge on fundamental market principles combined with this much richer feature set yields a clear competitive advantage over other sentiment analysis models.

Since CrudeBERT outperforms FinBERT, further refinements have been incorporated into the model. Considering background knowledge on the scope of market events, particularly by (i) identifying market entities such as countries and companies, and providing the model with information on their absolute and relative importance, as well as (ii) processing numerical clues paved the way for further improving CrudeBERT’s performance. Combining this information with social knowledge from Google Trends provided insights into an event’s impact and coverage, and yielded the CrudeBERT+ models.

The impressive performance of these models suggests that even a basic understanding of fundamental market principles considerably benefits Transformer models since it helps in better contextualizing the analyzed content.

For cumulative sentiment scores, CrudeBERT+ manages to outperform its competitors for most individual yearly periods as well as over the entire investigated 9-year period.

In terms of economic models, this paper focuses on Adam Smith’s law of supply and demand, since the investigated media articles have been dominated by events that can be properly explained by this law. Future work will aim at increasing the system’s predictive capabilities by extending its repertoire of supported economic models, covering new kinds of events such as changes in inflation and interest rates.

We also plan to draw upon domain experts from the financial industry to better quantify the impact of entities on commodity markets. Letting domain experts rank the effect of synthesized events and entities is expected to trigger further improvements in the CrudeBERT+ models.

Enabling CrudeBERT+ and its variants to work on full articles rather than headlines is another interesting research venue since articles usually provide more details on an event’s context and possible market impact. We, therefore, plan to prepare CrudeBERT+ models which are trained on the full article content.

Finally, we aim at developing methods capable of distinguishing between news articles that describe events relevant to future market movements (e.g., provide novel information to the market), and descriptive articles that reflect on recent market developments (e.g., contain new perspectives on known events). Distinguishing these two article types will be another important cornerstone for better estimating an article’s impact and temporal scope.

## Data Availability

The dataset created for and described in this research paper is publicly available at the following GitHub address: https://github.com/fhgr/crudeoil-sentiment2022-dataset.

## Footnotes

1 https://worldpopulationreview.com

2 https://www.sec.gov/edgar.shtml

3 https://sites.google.com/view/fiqa/home

4 https://ravenpack.com/discovery/news_analytics_realtime/

5 https://investing.com/

6 https://worldpopulationreview.com/

7 https://wikipedia.org/

8 https://trends.google.com/

9 https://trends.google.com/

10 https://github.com/fhgr/crudeoil-sentiment2022-dataset
